# Resection of Kommerell’s diverticulum in an infant with prenatal diagnosis of right aortic arch

**DOI:** 10.1186/s40792-019-0726-2

**Published:** 2019-11-06

**Authors:** Kenji Suzuki, Takashi Sasaki, Shinobu Kunugi, Yoshio Shima, Ryuji Fukazawa, Akira Shimizu, Takashi Nitta

**Affiliations:** 1 Department of Cardiovascular Surgery, Nippon Medical School, 1-1-5 Sendagi, Bunkyo-ku, Tokyo, 113-8602 Japan; 2 Department of Cardiovascular Surgery, Graduate School of Medicine, Nippon Medical School, Tokyo, 113-8602 Japan; 3 Department of Analytic Human Pathology, Graduate School of Medicine, Nippon Medical School, Tokyo, 113-8602 Japan; 4 Department of Neonatal Medicine, Nippon Medical School Musashi Kosugi Hospital, Kawasaki, Kanagawa 211-8533 Japan; 5 Department of Pediatrics, Graduate School of Medicine, Nippon Medical School, Tokyo, 113-8602 Japan

**Keywords:** Vascular ring, Prenatal diagnosis, Right aortic arch, Kommerell’s diverticulum, Tracheal compression

## Abstract

**Background:**

A right aortic arch is a congenital vascular anomaly that is present in up to 0.1% of pregnancies. The anomaly observed by fetal ultrasonography was recently reported to indicate vascular and chromosomal abnormalities that may complicate postnatal management.

**Case presentation:**

We report the successful resection of a Kommerell’s diverticulum with left subclavian artery transfer to the left carotid artery in a 5-month-old Japanese boy. The patient was prenatally diagnosed as having a right aortic arch, and a vascular ring was confirmed at 4 months of age with enhanced computed tomography. The pathology of the resected aortic wall revealed severe disruption and fragmentation of elastic fibers associated with a disarray of smooth muscle cells in the tunica media, and cystic medial necrosis with mucoid extracellular matrix deposition.

**Conclusion:**

These abnormal pathological findings supported the resection of Kommerell’s diverticulum at this point of time, and division of the ligamentum arteriosus alone was not recommended. Early intervention in this condition once the diagnosis is made may thus be advocated. The fetal diagnosis of a right aortic arch may provide a clue to the possibility of a vascular ring.

## Background

Right aortic arch (RAA) is a congenital vascular anomaly that is present in up to 0.1% of pregnancies. A RAA observed by fetal ultrasonography was recently reported to indicate vascular and chromosomal abnormalities that may complicate postnatal management [1]. We treated an infant with a symptomatic vascular ring who was diagnosed prenatally as having an RAA. Here, we review the patient’s clinical course and the surgical procedures.

## Case presentation

**Fig. 1:**
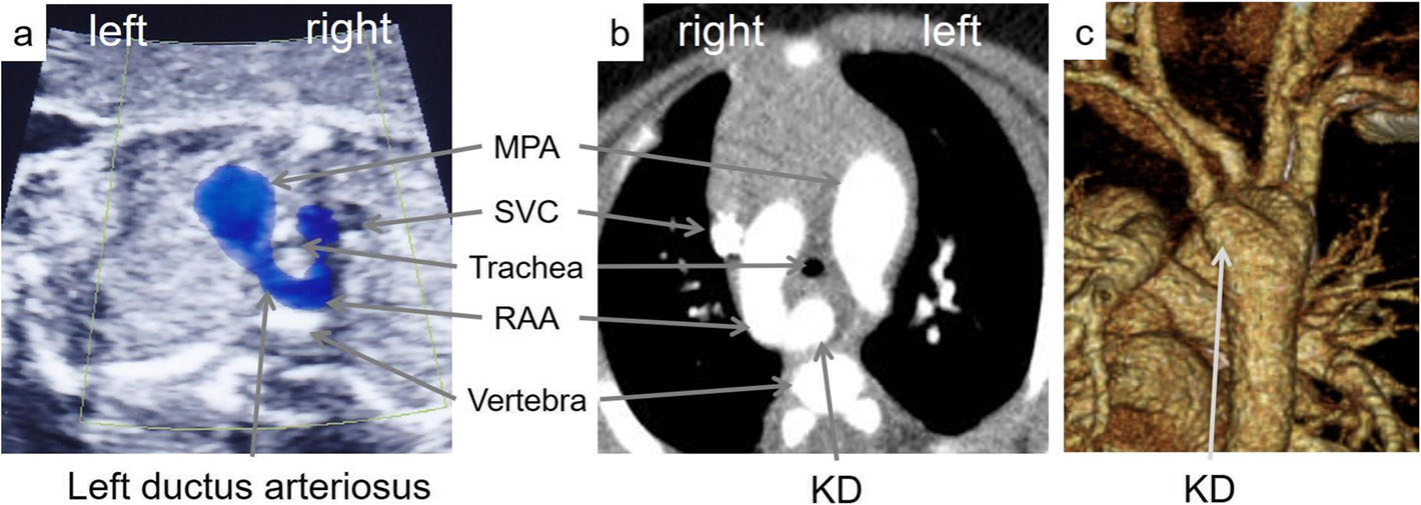
Prenatal ultrasonography and chest CT at preoperative status describing the RAA, retroesophageal LSCA, and KD leading to tracheal compression. **a** Prenatal ultrasonography from the upper mediastinum. **b** Horizontal image in chest CT. **c** 3D image from the left back in chest CT. KD: Kommerell’s diverticulum, LSCA: left subclavian artery, MPA: main pulmonary artery, RAA: right aortic arch, SVC: superior vena cava

A 4-month-old Japanese boy was referred to our institute for stridor that had been present since he was 2 months of age. Prenatal ultrasonography had revealed an RAA but no structural heart disease (Fig. 1). He was born at 38 weeks with a birth weight of 3066 g. At 2 months of age, he suffered from bronchitis and was admitted to the hospital; since then, he had shown stridor. A fiber laryngoscopy examination revealed no findings of congenital laryngeal stridor.

Computed tomography (CT) revealed an RAA, a retroesophageal left subclavian artery (LSCA), and Kommerell’s diverticulum (KD) leading to tracheal compression (Fig. 1). No intracardiac anomaly was detected by transthoracic echocardiography. Vascular ring repair was performed when the patient was 5 months old.

Under general anesthesia, bronchoscopy showed pulsatile compression from the right and left sides of the distal trachea. With the patient in a right lateral decubitus position, a muscle-sparing thoracotomy was performed. The chest was entered through the third intercostal space. The ligamentum arteriosum was divided. The 12-mm-diameter KD was resected, and the ostium on the descending aorta was closed with a running suture in two layers under the aortic partial clamp. The LSCA was transected at its origin from the KD and was anastomosed to the left carotid artery.

Once the left carotid artery was cross-clamped, the regional oxygen saturation in the left cerebral hemisphere was ~ 20% lower than that on the right side, and the lowest value was ~ 40%. Hypoventilation with high-concentration oxygen was then applied to achieve hypercapnia during the LSCA anastomosis. At the end of the surgery, bronchoscopy revealed that the pulsatile compression of the distal trachea had disappeared.

**Fig. 2:**
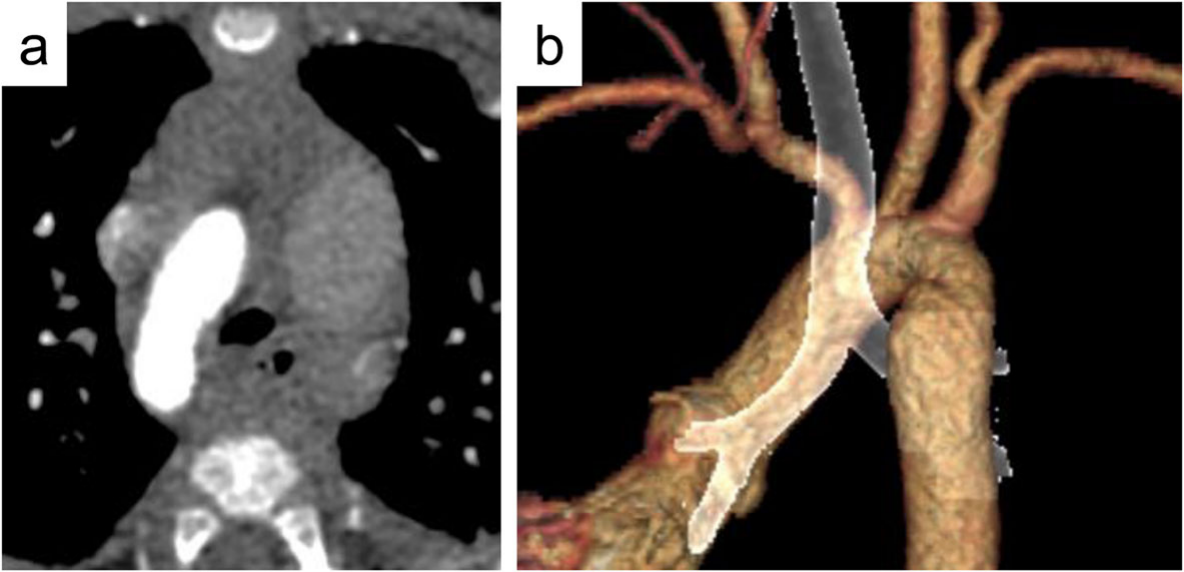
Chest CT at postoperative status describing the release of tracheal compression. **a** Horizontal image in chest CT. **b** 3D image from the left back in chest CT

Postoperative CT demonstrated the release of tracheal compression (Fig. 2). The patient was discharged 12 days after the operation, with no complications. At 1 year after the operation, the patient was asymptomatic, and no right-left difference in the blood pressure of the upper extremities was detected.

**Fig. 3:**
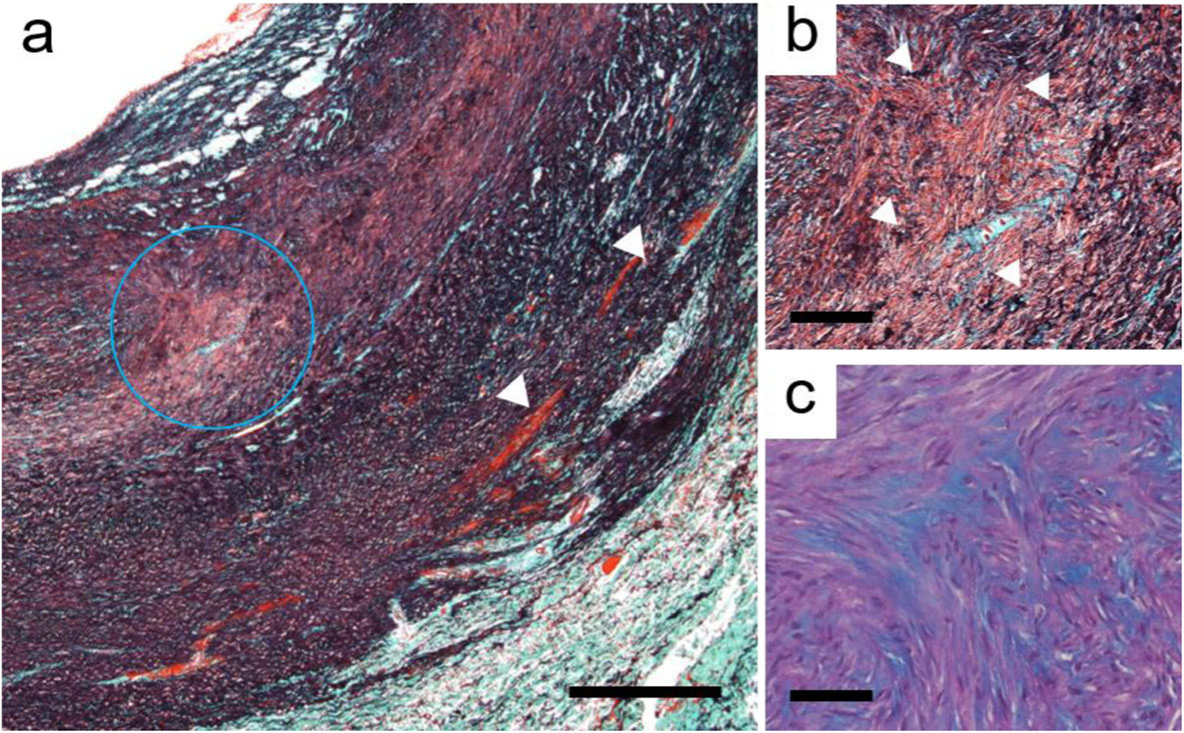
Pathological examination of the resected KD. **a** Low-magnification image showing severe disruption and fragmentation of elastic fibers (white arrowhead) associated with a disarray of smooth muscle cells. **b** High-magnification image of circled area in **a** showing the same findings. **c** High-magnification image of circled area in **a** showing cystic medial necrosis with mucoid extracellular matrix deposition. **a**, **b** Elastica-Masson-Goldner stain. **c** Alcian blue-Periodic acid-Schiff stain. **a** Bar = 500 μm. **b**, **c** Bar = 100 μm

The pathological examination of the resected KD revealed a marked difference in the arterial wall thickness between the aortic side and the distal side, severe disruption and fragmentation of elastic fibers associated with a disarray of smooth muscle cells in the tunica media, and cystic medial necrosis with mucoid extracellular matrix deposition (Fig. 3).

## Discussion

An aberrant LSCA arising from an RAA is present in approx. 0.05–0.10% of the population [2]. A retroesophageal LSCA with a left ligamentum arteriosum is called a true vascular ring [3]. An aneurysm-like change is often detected at the base of the aberrant LSCA from the RAA. This change was first described by Burckhard Kommerell in 1936 [4] as a KD, which is a remnant of the distal fourth aortic arch and a cause of tracheal and esophageal compression [3] symptoms such as dysphagia, shortness of breath, and chest pain.

There are two types of surgery for a vascular ring in RAA with aberrant LSCA. One is the division of the ligamentum arteriosus, resection of the KD, and LSCA transfer to the left carotid artery; these were performed in our patient’s case. The other type of surgery is merely dividing the ligamentum arteriosus with arteriopexy [5]. If arteriopexy is not performed in this method, recurrent tracheal compression may occur because of the large size of the KD.

The abnormal pathological findings of the resected aortic wall of our patient’s KD supported the resection of the KD itself. A concern was the right-left difference in the patient’s cerebral regional oxygen saturation during the LSCA anastomosis to the left carotid artery; however, no neurological symptoms have been detected up to the present. Moreover, patients who have undergone merely a division of the ligamentum arteriosus tend to have postoperative respiratory complications. Shinkawa et al. noted three of eight patients with only a division of the ligamentum arteriosus had mild asthma-like respiratory symptoms and received albuterol aerosol or oral anti-inflammatory medication; however, all 10 of their patients with a primary translocation of an aberrant LSCA with diverticulum removal and ligamentum division remained free from residual symptoms or medications [6].

Interventions for a KD are a recent focus of discussion in adult cardiovascular surgery. Various procedures have been reported to treat a KD, including graft replacement of the descending aorta with the in situ reconstruction of the aberrant SCA, graft replacement of the descending aorta with a bypass to the aberrant SCA, and total arch replacement. The overall early mortality was 5.3% in elective cases [7]. Endovascular repair cannot attenuate the compressive symptoms caused by a vascular ring formation; in addition, the long-term results are still unknown [8]. Compared to vascular ring repair in infancy, the mortality of which was 0% in Japan from 2014 to 2016 [9–11], interventions for a KD in adulthood are much more complicated and high-risk procedures. From this point of view, the resection of a KD should be performed during infancy.

Regarding the fetal diagnosis of an RAA, the RAA of many patients is accompanied by a vascular ring after birth [12]. Vigneswaran et al. stated that pulsatile tracheal compression was identified under bronchoscopy in 94% of neonates with a prenatally diagnosed RAA and left patent ductus arteriosus, and the surgical relief of a vascular ring was needed in 79% of these cases at the median age of 15 months [13]. A radiological evaluation should be performed with enhanced CT as expeditiously as practicable. The compressive symptoms are mild in most cases, and surgery can be postponed until the patients become symptomatic. However, once the patient shows a compressive symptom such as stridor, the patient should undergo the division of the vascular ring soon, because it was reported that the early repair of a vascular ring might allow for normal development of the trachea and less postoperative respiratory complications (especially respiratory infections) [13]. In present case, his fetal diagnosis of RAA and stridor in his infancy leaded to CT imaging and surgical intervention at an appropriate time.

Continuous follow-up imaging such as CT is necessary in cases of KD. Aortic aneurysm [14] or aortic dissection [15] may develop, because mucoid medial degeneration with cystic necrosis has been observed in the internal part of a KD [16]. In the present case, we identified an anomaly by pathological examination at the edge of the resected tissue, and it is thus possible that adverse aortic events may occur in the future.

The pathological examination in our patient’s case revealed that the KD had a disarray of smooth muscle cells and a difference in the arterial wall thickness. This case can contribute to the elucidation of the process of the formation of a KD.

To date, this anomaly has been diagnosed after the development of tracheal and esophageal compression symptoms. The increasing use of prenatal ultrasonography will enable the diagnosis of an anomaly of aortic arch and branch vessels before the development of compression symptoms, and as a result, a greater number of surgeries will be performed to eliminate the risk of adverse aortic events. We do not have any data regarding the relationship between the size of a diverticulum and the risk of adverse aortic events; however, we suggest that asymptomatic patients with a small diverticulum should undergo thorough follow-up examinations, including CT.

## Conclusion

A 5-month-old boy with the prenatal diagnosis of a RAA had a vascular ring composed of a RAA, an aberrant LSCA, left-side ligamentum arteriosus, and a main pulmonary artery. He underwent the division of the left-side ligamentum arteriosus, resection of the KD, and the transfer of the LSCA to the left carotid artery. His tracheal compression symptom disappeared after the surgery. Pathology revealed abnormal findings on the resected aortic wall, and this supported the concomitant resection of the KD.

## Data Availability

The datasets supporting the conclusions of this article are included within the article.

## Abbreviations

CT: Computed tomography
KD: Kommerell’s diverticulum
LSCA: Left subclavian artery
RAA: Right aortic arch

## References

[1] Razon Y, Berant M, Fogelman R, Amir G, Birk E (2014). Prenatal diagnosis and outcome of right aortic arch without significant intracardiac anomaly. J Am Soc Echocardiogr.

[2] Cina CS, Althani H, Pasenau J, Abouzahr L (2004). Kommerell’s diverticulum and right-sided aortic arch: a cohort study and review of the literature. J Vasc Surg.

[3] Backer CL, Monge MC, Popescu AR, Eltayeb OM, Rastatter JC, Rigsby CK (2016). Vascular rings. Semin Pediatr Surg.

[4] Kommerell B (1936). Verlagerung des osophagus durch eine abnorm verlaufende arteria subclavia dextra (arteria lusoria). Fortschr Geb Rontgenstr.

[5] Naimo PS, Fricke TA, Donald JS, Sawan E, d'Udekem Y, Brizard CP (2017). Long-term outcomes of complete vascular ring division in children: a 36-year experience from a single institution. Interact Cardiovasc Thorac Surg.

[6] Shinkawa T, Greenberg SB, Jaquiss RD, Imamura M (2012). Primary translocation of aberrant left subclavian artery for children with symptomatic vascular ring. Ann Thorac Surg.

[7] Tanaka A, Milner R, Ota T (2015). Kommerell’s diverticulum in the current era: a comprehensive review. Gen Thorac Cardiovasc Surg.

[8] Goto Y, Koyama Y, Hosoba S, Ogawa S, Fukaya S, Okawa Y (2018). Total arch replacement through a median sternotomy for Kommerell’s diverticulum. Asian Cardiovasc Thorac Ann.

[9] Masuda M, Okumura M, Doki Y, Endo S, Hirata Y, Committee for Scientific Affairs TJAfTS (2016). Thoracic and cardiovascular surgery in Japan during 2014 : annual report by the Japanese Association for Thoracic Surgery. Gen Thorac Cardiovasc Surg.

[10] Masuda M, Endo S, Natsugoe S, Shimizu H, Doki Y, Committee for Scientific Affairs TJAfTS (2018). Thoracic and cardiovascular surgery in Japan during 2015: annual report by the Japanese Association for Thoracic Surgery. Gen Thorac Cardiovasc Surg.

[11] Shimizu H, Endo S, Natsugoe S, Doki Y, Hirata Y, Committee for Scientific Affairs TJAfTS (2019). Thoracic and cardiovascular surgery in Japan in 2016: annual report by the Japanese Association for Thoracic Surgery. Gen Thorac Cardiovasc Surg.

[12] Campanale CM, Pasquini L, Santangelo TP, Iorio FS, Bagolan P, Sanders SP, et al. Prenatal echocardiographic assessment of right aortic arch. Ultrasound Obstet Gynecol. 2019;54:96–102. 10.1002/uog.20098 30125417

[13] Vigneswaran TV, Kapravelou E, Bell AJ, Nyman A, Pushparajah K, Simpson JM, et al. Correlation of symptoms with bronchoscopic findings in children with a prenatal diagnosis of a right aortic arch and left arterial duct. Pediatr Cardiol. 2018;39:665–73. 10.1007/s00246-017-1804-5 29307026

[14] Fisher RG, Whigham CJ, Trinh C (2005). Diverticula of Kommerell and aberrant subclavian arteries complicated by aneurysms. Cardiovasc Intervent Radiol.

[15] Braunberger E, Mercier F, Fornes P, Julia PL, Fabiani JN (1999). Aortic dissection of Kommerell’s diverticulum in Marfan’s syndrome. Ann Thorac Surg.

[16] Luciano D, Mitchell J, Fraisse A, Lepidi H, Kreitmann B, Ovaert C (2015). Kommerell diverticulum should be removed in children with vascular ring and aberrant left subclavian artery. Ann Thorac Surg.

